# Feasibility, efficacy, and safety of animal-assisted activities with visiting dogs in inpatient pediatric oncology

**DOI:** 10.1007/s12519-024-00829-8

**Published:** 2024-08-07

**Authors:** Katja Steff, Maximilian Grasemann, Kira Ostermann, Sarah Christina Goretzki, Peter-Michael Rath, Dirk Reinhardt, Michael M. Schündeln

**Affiliations:** 1 Pediatric Hematology and Oncology, Department of Pediatrics III, University of Duisburg-Essen, Hufelandstr. 55, 45122 Essen, Germany; 2 Department of Psychology, Medical School Hamburg, Hamburg, Germany; 3 Pediatric Infectiology, Department of Pediatrics I, University of Duisburg-Essen, Essen, Germany; 4 Institute of Medical Microbiology, University of Duisburg-Essen, Essen, Germany

**Keywords:** Animal-assisted activities, Animal-assisted interventions, Animal-assisted therapy, Feasibility, Infection control, Inpatient pediatric oncology, Safety, Stress response, Therapy dog, Visiting dog

## Abstract

**Background:**

Childhood cancer entails a heavy burden for patients and their families. Recent advances in overall survival rates have increasingly brought long-term quality of life into focus. Animal-assisted activities (AAAs) have long been hypothesized to alleviate the burden on pediatric patients and their peers in the hospital setting. However, their use in inpatient pediatric oncology has been a sensitive issue mainly due to the fear of infections, resulting in a lack of studies. This study presents data on the feasibility, safety, and efficacy of AAAs from a single German center.

**Methods:**

Between 2018 and 2022, 60 patients (median age = 10.3 years) diagnosed with malignancy and undergoing treatment were visited by an intervention dog (total visits = 100). Patients were screened for infections as per hospital policy, with additional microbiological testing performed based on symptoms. The dog was screened for human pathogens and zoonoses. Microbial data and hospitalizations were analyzed from two months prior to the first visit until two months after the last visit. Acceptance of being in the hospital, both with and without planned animal-assisted interventions and pre- and post-intervention state stress, were measured using a validated visual analogue scale (0–10).

**Results:**

Patients benefited from AAAs, showing increased acceptance of being in the hospital (median: 7.25 vs. 4.50, *P* < 0.001) and decreased median state stress ratings one hour after the visit compared to one hour before the visit (1.00 vs. 4.25, *P* < 0.001). The intervention did not result in an increased number of infections or unplanned hospitalizations, and no zoonoses were detected. All microbial screening tests of the dog were negative.

**Conclusions:**

AAAs with visiting dogs in inpatient pediatric oncology are feasible and safe. Although they hold promise for enhancing patients’ well-being, further prospective studies are needed.

Supplementary file 2 (MP4 240076 KB)

**Supplementary Information:**

The online version contains supplementary material available at 10.1007/s12519-024-00829-8.

## Introduction

The diagnosis of cancer places a heavy burden on patients and their families and presents challenges for healthcare providers. This holds particularly true for children and adolescents with malignancies [1]. Patients are often isolated from their peers, while parents worry about their children’s health and siblings struggle with the new order and prioritization of family life. Although most children and adolescents with cancer can be cured today [2], the disease and its treatment result in notable acute and chronic psychosocial challenges for both the child and their family [3, 4].

More complex, intense, and patient-adapted precision medicine has led to improvement in the overall survival of patients [5]. Therefore, there has been an increasing focus on the long-term sequelae and quality of life of these survivors in recent decades [6, 7]. There is also a growing recognition of the potential benefits of complementary therapies to enhance the overall well-being of children undergoing cancer treatment [8, 9].

In this context, animal-assisted interventions (AAIs) have long been hypothesized to alleviate the burden on patients and their peers in the hospital setting. ﻿Both animal-assisted therapy (AAT) and animal-assisted activities (AAAs) are forms of AAIs in which the animal may be part of a volunteer therapy animal team working under the guidance of a professional, or belonging to a professional [10]. While AAT is an intervention in which the animal is a defined part of the treatment process, AAA is not goal-directed but provides opportunities for motivation, recreation, and enhanced quality of life.

AAIs in pediatric patients have been shown to alleviate pain [11] and improve well-being [12]. In a recent systematic review, Correale et al. describe the biobehavioral response to stress and pain, social behavior, quality of life, and level of satisfaction with hospitalization in children and adolescents [13]. The positive impact of AAIs has been widely acknowledged in various healthcare contexts. However, their potential in the pediatric oncology inpatient settings is a subject of increasing interest and ongoing research. The introduction of AAAs into the pediatric oncology setting is a sensitive issue, particularly due to concerns about microbial sharing between pediatric oncology patients and therapy dogs [14]. The additional risk of introducing infections and zoonoses to this vulnerable population remains a subject of ongoing debate [15, 16]. Despite the sparsity of studies in this field, most of the top-ranked US hospitals have established AAI programs in their pediatric oncology units [17]. In Germany, the number of hospitals with experience in animal interventions remains lower than in other countries, with established animal visit programs in only about one-third of institutions [18].

This study was conducted as a single-center pilot study with 100 animal visits to assess the safety and feasibility of AAIs in the inpatient pediatric oncology setting in Germany and to generate data on their efficacy regarding well-being and stress relief. Based on the data generated from this study, a prospective confirmatory study is being designed to shed light on the nuanced dynamics of AAIs in pediatric oncology and potentially improve short- and long-term quality of life in this population.

## Methods

### Patients

The study was conducted between 2018 and 2022 in a large tertiary center in Germany. Inclusion criteria were admission to the inpatient oncology ward, a diagnosis of childhood hematologic or solid malignancy, written informed consent, and the absence of exclusion criteria. Exclusion criteria were allergies to dogs, cynophobia, severe neutropenia, fresh or uncoverable wounds, bacterial infections, or colonization with nosocomial pathogens. Patients who fulfilled these criteria were offered study participation by the attending pediatric hematology-oncology specialist. The databank was closed on July 1, 2023.

### Dog/interventions

To minimize potential risks posed by the AAIs, a written safety and hygiene policy was developed prior to starting the program. The policy was developed largely following the recommendations from the Society of Healthcare Epidemiology of America (SHEA) [19]. All AAAs were conducted by a male Labrador retriever named Hannibal, aged 7–11 years. Hannibal was first trained as a search and rescue dog at the age of 24 months and received additional training as a therapy dog at the age of 6 years. His training and certification were performed at an institution accredited by the International Society for Animal Assisted Therapy [20]. The dog was owned by a volunteer medical doctor and a volunteer paramedic, both of whom were trained as specialists for AAIs and alternately guided the AAA sessions. The handlers were not part of the core medical team but were well-informed on the specific processes of a pediatric oncology department. The dog/handler team underwent annual recertification. The intervention was not designed to follow a defined goal or schedule. Therefore, Hannibal acted as a visiting dog [21] in an AAA setting. AAA sessions were offered once weekly, lasting between 25 and 45 minutes. Supervised by the handler, the patient and parents decided the level of interaction they preferred, ranging from passive to active. Passive interaction involved the dog sitting or sleeping next to the patient and active interaction included playful roughhousing and pet tricks.

### Safety, infection control procedures, and hygiene practices

Before engaging in contact with the visiting dog, a history of allergy to dogs was ruled out for patients and accompanying persons. Patients with a fear of dogs were offered to participate and gently introduced to the situation, with the option of being able to leave the room at any point. None of the patients made use of this option. Patients with neutropenia (< 500/µL), recently operated patients (< 7 days) after any kind of surgery, and patients unable to cover long-lasting wounds were excluded during those periods. Patients with known colonization with Methicillin-resistant *Staphylococcus aureus* (MRSA), multi-resistant Gram-negative bacteria (MRGN), or known bacterial infections were excluded until cleared from colonization or infection. Patients and handlers were required to sanitize their hands before and after each visit to prevent the spread of pathogens between dog, handler, and patients. They were also advised to change clothes after the intervention, and all contact areas in the room were cleaned and disinfected as recommended by SHEA [19].

To guarantee the safety of the patient and dog, the handler was advised to interrupt the visit at any time if the development of interaction was assessed as dangerous. In addition, a retreat place with a dog blanket was defined in which Hannibal would not be disturbed. He had the option to leave the setting at any time and seek out his retreat. Neither of these measures had to be implemented at any time.

With each admission, patients were tested for MRSA using a pooled nasal-pharyngeal swab. Swabs were also obtained from all wounds and stomata. Patients were tested for MRGN routinely upon first admission. Additional testing was performed if they had a recent history of hospitalization outside of Germany, known prior MRGN status, history of contact with an MRGN-positive individual, or if they were transferred from the intensive care unit. Stool tests for bacteria and rectal swabs were also performed routinely upon first admission.

Outside of these indications, microbiological testing was performed based on clinical symptoms and the judgment of the treating medical team. Hannibal was dewormed regularly, at least every six months, and seen by a veterinarian twice yearly. His vaccinations included hepatitis contagiosa canis, leptospirosis, canine distemper, and rabies. Hannibal was routinely tested every four weeks during the trial for parasites and pathological bacteria by collecting stool, rectal, and snout swabs.

To assess the potential risk of infections or other adverse events (AEs) that may have been induced by contact with the visiting dog, the following data for each patient were collected: (1) all hospitalizations starting from the first visit until two months after the final visit; (2) all scheduled hospitalizations for therapy during this time; (3) all inpatient episodes for fever/sepsis; (4) all AEs as defined by the Common Terminology Criteria for Adverse Events (version 5.0); and (5) all hospitalizations for other reasons. The observation period for each patient spanned from two months prior to the first dog contact to two months after the last dog contact. In total, 3510 days (observed patient days) were monitored prior to the first visit and 4832 days were monitored after the first visit until two months after the last. Four patients had a shorter observation period (15–35 days) prior to the first visit and thus did not have complete pre-visit microbial screening data.

### Efficacy measures

To measure the efficacy of the intervention, validated visual analogue face rating scales [22] were used as a quick and easy-to-use low-barrier approach. Upon admission, the patients were asked to rate their acceptance of being at the hospital on that day (0 = I am not happy at all to be here, 10 = I enjoy very much being here). For patients with a scheduled dog visit, this question was asked before meeting the visiting dog. A similar visual analogue scale was used to rate the patients’ level of state stress (0 = no stress at all, 10 = maximum distress) prior to and one hour after the intervention. For patients with multiple visits, the median result was used for analysis. For patients who were unable to answer these questions, ratings given by their parents were used as an external assessment. After the dog visit, a brief, non-structured post-intervention interview was held with the patients and their caregivers. Immediately after the AAAs, patients and parents were asked to spontaneously reflect on their impressions of the intervention. The dog handler acted as the interviewer, following this narration and generating questions based on it.

### Statistics

Statistical analyses were performed with the R base/stats package (R Core team, 2020). PRISM for MAC 7.0 (GraphPad Software, Inc., La Jolla, CA, USA [23]) and R base package ggplot2 [24] was used to generate figures. Values are expressed as the mean ± standard deviation, median, and range unless stated otherwise. In most of the variables, normal distribution could not be assumed. Therefore, associations between single variables were described by the Spearman correlation coefficient. Differences in continuous variables between the groups were tested using the Wilcoxon rank-sum test for two-group comparisons. Multiple testing was not corrected in this hypothesis-generating, non-confirmatory study. Group differences in categorical variables were tested using the Chi-square test. For all tests, statistical significance was presumed at *P* < 0.05.

### Ethics

The study was performed in accordance with the ethical principles of the Declaration of Helsinki and with the approval from the local research ethics committee (20-9153-BO). Informed consent to participate in this study was provided by the participant’s legal guardian/next of kin and eligible patients. The recommendations for animal welfare by the International Association of Human–Animal Interaction Organizations were followed [25].

## Results

### Clinical characteristics

The cohort consisted of 60 patients (29 female). The median age at visit was 10.3 years. There were a total of 100 visits, with the number of visits per patient ranging from one to five (median: one). The mean timespan between the first and last dog visit was 20.5 days (1–211 days) (Table 1).

**Table 1:** Clinical characteristics of the patients

Variables	Male	Female	Overall
Sex, *n* (%)	31 (52)	29 (48)	60
Age at first visit (y)			
Mean (SD)	9.67 (4.09)	11.2 (4.51)	10.4 (4.33)
Median (min, max)	9.70 (2.07, 18.0)	12.10 (3.47, 17.9)	10.70 (2.07, 18.0)
Number of dog-visits			
Total	43	57	100
Median (min, max)	1.00 (1.00, 4.00)	1.00 (1.00, 5.00)	1.00 (1.00, 5.00)
Time between first and last dog visit (d)			
Mean (SD)	14.4 (34.2)	27.1 (50.3)	20.5 (42.9)
Median (min, max)	1 (1, 146)	1 (1, 211)	1 (1, 211)
Follow-up after first dog visit (d)			
Mean (SD)	1460 (600)	1630 (556)	1540 (581)
Median (min, max)	1580 (85, 2150)	1850 (357, 2160)	1720 (85, 2160)
Diagnosis/category, *n* (%)			
Acute lymphoblastic leukemia	10 (32.3)	8 (27.6)	18 (30.0)
Sarcoma	4 (12.9)	9 (31.0)	13 (21.7)
Other hematologic malignancy	6 (19.4)	6 (20.7)	12 (20.0)
Embryonal malignancy	5 (16.1)	3 (10.3)	8 (13.3)
Other malignancy	3 (9.7)	2 (6.9)	5 (8.3)
Malignant brain tumor	3 (9.7)	1 (3.4)	4 (6.7)

*SD* standard deviation

### Patient safety

To quantify patient safety and risk, we documented all hospital visits two months prior to the first AAI and two months after. We also followed up with patients until data bank closure. The median follow-up of the overall cohort from the initial dog visit to data bank closure or death of the patient was 1720 days. Five patients died during the follow-up period, all from progressive disease (85–507 days after the last visit). No patients died from infections or non-cancer-related reasons.

A total of 154 hospitalizations were counted prior to the first intervention, and 148 hospitalizations were recorded after the first intervention. Hospitalizations due to fever and infections were 34 and 31, respectively. This corresponds to an incidence density of 0.97 per 100 observation days (95% CI = 0.64–1.35) prior to the first visit and 0.64 per 100 observation days (95% CI = 0.44–0.91) after the first visit. Other AEs were found in 22 cases before the visit and 21 cases after the visit. None of the observed numbers differed significantly between the periods before and after the visit, as assessed by the Wilcoxon rank-sum test (Table 2). The detailed summary data regarding microbiology testing is given in Table 3. The presentation of microbial pathogen findings is displayed in Table 4.

**Table 2:** Hospitalizations of the observed patients before and after the first intervention

Variables	2 mon prior to the first visit				Until 2 mon after the last visit				*P* (Wilcoxon rank-sum test)
	Sum	Median	Min	Max	Sum	Median	Min	Max	
Total	154	3	1	6	148	2	1	8	0.780
Fever	34	0	0	3	31	0	0	3	0.740
Averse event	22	0	0	3	21	0	0	4	0.910
Other	11	0	0	2	16	0	0	2	0.357
Therapy	102	2	0	5	92	1	0	5	0.531

Overall sum, range, median, and range of hospitalizations of 60 observed patients are given from two months before the first visit and from the first visit until two months after the last dog visit

**Table 3:** Microbiology of the observed patients before and after dog visit

Test	2 mon prior to the first visit			Until 2 mon after the last visit			*P* (Chi-square test)
	Tested patients	Positivepatients	Total tests	Tested patients	Positive patients	Total tests	
Bacterial swabs							
Nasal-pharynx	56	0	171	60	2	163	0.172
Anal/genital	24	2	37	17	3	23	0.369
Other (wounds, etc.)	11	1	16	11	4	23	0.127
Stool							
Culture	56	1	77	31	1	38	0.685
Clostridioides difficile toxin	56	4	77	31	1	38	0.434
Clostridioides difficile antigen	56	4	77	31	5	38	0.188
Campylobacter antigen	56	0	77	31	0	38	NA
Serum/blood							
Mycological screening	31	8	120	31	3	80	0.108
Blood culture	37	6	167	34	7	122	0.634
Septifast®	9	1	20	2	1	10	0.197
Virology	39	8	180	29	6	136	0.986
Urine							
Culture	23	2	51	19	4	49	0.255
Other material							
Other	6	1	46	16	1	72	0.449

Number of tested patients, number of positive tests and the total number of tests of 60 observed patients are given from two months before the first visit and from the first visit until two months after the last dog visit. *NA* not applicable

**Table 4:** Detailed presentation of positive microbial findings before and after dog visit

Test	2 mon prior to first visit	Positive tests	Positive patients	Until 2 mon after last visit	Positive tests	Positive patients
Nasopharyngeal bacterial swabs	–	0	0	*S. aureus*	1	2
				*M. pneumoniae*	1	
Anorectal swabs	*E. coli*	1	2	*E. faecalis*	1	3
	*K. pneumoniae*	1		*C. albicans*	1	
				*P. aeruginosa*, 4 MRGN	1	
				*E. coli*	2	
Other swabs	*S. aureus* (foot)	2	2	*E. coli* (genital/tongue)	2	3
				*Acinetobacter* spp., 4 MRGN (hands)	1	
				*S. aureus* (wound)	1	
Stool cultures	*C. difficile* toxin	1	1	*C. krusei/C. albicans*	1	1
Mycology screening	1–3-beta-D-glucan test positive	7	8	1–3-beta-D-glucan test positive	3	3
	*Candida* antigen	2		*Candida* antigen	1	5
Positive blood cultures	*S. epidermidis*	3	6	*S. epidermidis*	4	
	*E. coli*	2		*M. luteus*	1	
	*B. cereus*	1		*P. aeruginosa*	1	
				*E. fergusonii*	1	
RT-PCR	*K. pneumoniae*	1	1	*P. aeruginosa*	1	1
Virology findings	CMV IgM	1	7	Influenza A RNA	3	7
	HSV1 DNA	2			1	
	Parainfluenza	3		Influenza B RNA	2	
	Rhinovirus RNA	1		Rhinovirus RNA	2	
	Adenovirus DNA	1		HSV1 DNA		
Urine cultures	*E. faecium*	1	2	*C. albicans*	2	4
	*E. coli*	1		*E. coli*	1	
				*E. faecalis*	1	
Other material	*S. epidermidis* (catheter tip)	1	1	*S. epidermidis* (catheter tip)	1	1

Number of positive tests and total number of positive patients for each category are given from two months before first visit and from first visit until two months after last dog visit. *RT-PCR* real-time polymerase chain reaction (ROCHE SeptiFast®), *CMV* cytomegalovirus, *IgM* immunoglobulin M, *HSV1* herpes simplex virus 1, *MRGN* multiresistant Gram-negative bacteria, *S*. *aureus Staphylococcus aureus*, *M. pneumoniae Mycoplasma pneumoniae*, *E. faecalis Enterococcus faecalis*, *C. albicans Candida albicans*, *P. aeruginosa Pseudomonas aeruginosa*, *E. coli Escherichia coli*, *K. pneumoniae Klebsiella pneumoniae*, *C. difficile Clostridioides difficile*, *C. krusei Candida krusei*, *S. epidermidis Staphylococcus epidermidis*, *M. luteus Micrococcus luteus*, *E. fergusonii Escherichia fergusonii*, *B. cereus Bacillus cereus*, *E. faecium Enterococcus faecium*

### Dog, safety, and microbiology

Hannibal was tested for infections and colonization regularly (Table 5). The results of testing for pathogenic bacteria and colonization with MRSA, MRGN, and vancomycin-resistant enterococci were negative in all cases throughout the course. On one occasion *Campylobacter* infection was suspected after a positive *Campylobacter* antigen enzyme immunoassay (EIA). However, it was ruled out by negative DNA testing for *Campylobacter* spp. and was therefore interpreted as an unspecific reaction of the EIA.

**Table 5:** Four-weekly testing of therapy dog for pathogens

Test	Pathogen	Material
Bacterial cultures	*Escherichia coli*	Stool
	*Salmonella* spp.	Stool
	*Shigella* spp.	Stool
	*Yersinia* spp.	Stool
	*Campylobacter* spp.	Stool
	MRGN *Enterobacter* spp.	Rectal swab
	MRGN *Acinetobacter baumanii*	Rectal swab, snout swab
	MRGN *Klebsiella pneumoniae*	Rectal swab
	MRSA	Snout swab
	VRE	Rectal swab
Toxin tests (PCR)	Shigatoxin *(E. coli*)	Stool
	Verotoxin (*E. coli*)	Stool
Antigen tests (EIA)	*Campylobacter* spp.	Stool
	*Entamoeba histolytica*	Stool
	*Giardia intestinalis*	Stool
Direct microscopy	Helminths (eggs/larvae/adults)	Stool
	*Amoeba* spp.	Stool

Microbial testing for pathogens was performed regularly by culture, toxin test, antigen test and microscopy. *MRGN* multiresistant gram-negative bacteria, *MRSA* methicillin-resistant *Staphylococcus aureus*, *VRE* vancomycin-resistant enterococci, *PCR* polymerase chain reaction, *EIA* enzyme immunoassay

### Efficacy measures

**Fig. 1:**
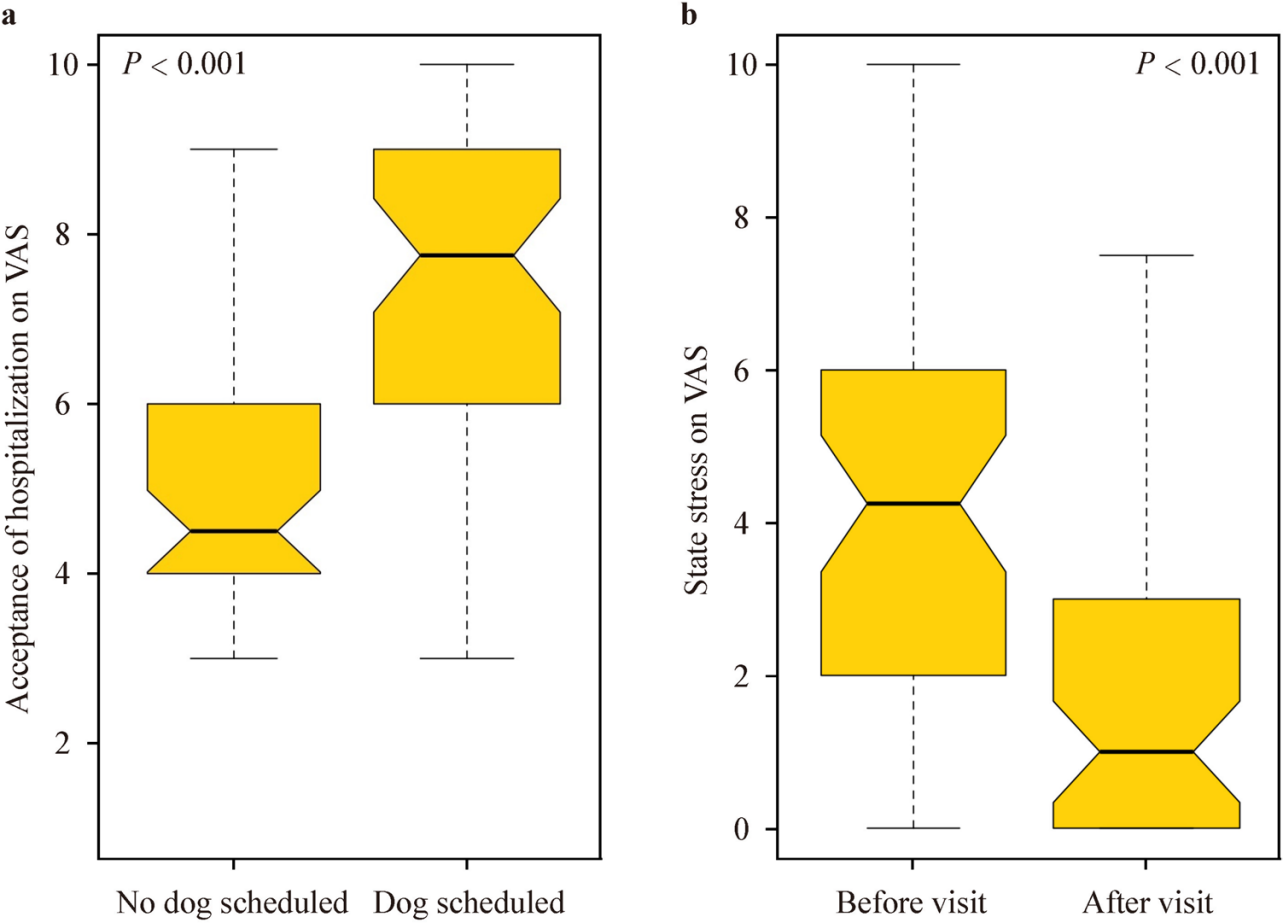
The self-rating of patients. **a** Patients with or without planned animal-assisted therapy intervention were asked how much they enjoyed coming to the hospital; **b** patients with dog visits were asked to rate the level of state stress one hour before and one hour after the visit. The box depicts 25th to 75th percentile and median. Whiskers represent all samples lying within 1.5 times the IQR. *VAS* visual analogue scale, *IQR* interquartile range

The study also aimed to assess the patients' comfort level and stress levels in the hospital. The patients were asked on admission to rate their acceptance of being at the hospital. Patients who knew that they were scheduled to meet Hannibal had significantly higher scores on a scale from 0 to 10 than patients who had no scheduled dog visit [7.75 (0–10), *n* = 50 vs. 4.5 (3–9), *n* = 43, *P* < 0.001] (Fig. 1a). Patients who were asked about their state stress level on a scale from 0 to 10 responded with significantly lower ratings one hour after the visit than one hour before the visit [1 (0–7.5), *n* = 50 vs. 4.25 (0–10), *n* = 51, *P* < 0.001] (Fig. 1b).

### Results from the post-interventional interviews

The most prominent subject of the children’s interview narratives after the intervention were pleasure and distraction: “I am glad, that I can play with him and pet him”. “It was very cool to play with Hannibal”. The parents´ interviews also revealed some more long-lasting subjects such as opening up and raising self-esteem: “This is the first time that my son has been laughing since diagnosing his leukemia”. “My daughter became much more talkative, brighter and was laughing much more following the intervention”. “The program makes a huge difference”. “He regained a lot of self-esteem”. “Please carry on with the program, even if it is a huge effort”. Meanwhile, the results of the non-structured interviews revealed no negative impressions in any of the cases.

## Discussion

This study adds proof that AAAs with visiting dogs are feasible, effective, and safe in an inpatient pediatric oncology setting in a German institution. Most evidence for the feasibility and efficacy of AAIs in the pediatric oncology setting stems from small, non-controlled studies. In 2004, Gagnon et al. [26] published a descriptive pilot study involving interviews with 16 parents of children with cancer, concluding that dog-assisted therapy may be feasible and that patients may benefit from it. Bouchard et al. [27] described an improvement in well-being in patients, parents, and pediatric oncology inpatient medical team in a pilot study with 27 patients. Chubak et al. [28] came to similar conclusions in a non-controlled pilot study with 19 patients. AAAs resulted in lower distress, tiredness, sadness, fear, and pain without added risks for the patients. In an exploratory study by Tsai et al. [29], nine hospitalized children and six controls were examined. The authors reported a decrease in systolic blood pressure during and minutes after the AAAs, which they interpreted as a possible decrease in the autonomic stress response. Anxiety and medical fear did not differ after AAAs when compared to the control group. Recently, small, randomized trials have been published in the pediatric oncology setting. Chubak et al. [30] were unable to detect a difference in psychological functioning between the control and intervention groups. However, their trial was halted due to the restrictions of the COVID-19 pandemic. With only 12 patients in the intervention group, the study was underpowered. McCullough et al. published data from a parallel-group multicenter, randomized trial with 60 patients in the intervention group. A reduction of state anxiety was detected in both the intervention and control groups, without a significant difference between them [31]. In addition, there were no group differences in changes over time for quality of life. Parental stress, however, was reduced significantly in the intervention group. The authors discuss that the study was underpowered due to the heterogenicity of the different hospital site settings. Branson et al. [32] measured positive and negative affect scores, as well as salivatory cortisol and C-reactive protein as stress biomarkers, before and after AAAs. The randomized controlled study incorporated 24 participants in both groups. Although the study showed changes in the expected direction, no significant differences were found, highlighting the risk of underpowered studies when designing a trial measuring highly volatile (bio-) markers. In summary, published studies on the efficacy of AAIs in pediatric oncology remain sparse and results are not always in line with the hypotheses. Feng et al. [33] reviewed the subject systematically in the general pediatric population, while Cotoc et al. [34] focused on the pediatric oncology population in their review.

The present study adds evidence towards the hypothesis of AAAs with visiting dogs being beneficial for the well-being of children in the pediatric oncology hospital setting. The visual analogue scale data indicate that the acceptance of hospitalization is higher when scheduling a visit with a therapy dog. This is particularly important as children often struggle to understand the need for frequent hospitalizations [35]. Furthermore, this study found that the level of state stress [32] was shown to be reduced significantly around the time of the AAIs. The unstructured interviews invariably approved of the intervention and indicated positive effects on well-being and patient pleasure throughout. Moreover, interview data from patients and parents with multiple visits suggests that longer-lasting effects of the intervention may be present.

AAA is a team approach, and its success depends significantly on the teamwork and coordination of the dog handler with the hospital team [36]. As such, members of the AAA team need a sound understanding of the local circumstances and processes in the respective institution. Of note, the effects of the interventions on the team were not in the scope of the study and were not systematically measured. Anecdotal records indicate that the possible positive effects of the dog visits on the well-being of the team should not be underestimated. The visiting dog was literally a door opener, with a regular statement from the medical and administrative team in the hospital being: “When is Hannibal coming again?”. In line with our observations, other groups have also revealed the psychological benefits of AAIs for healthcare workers [37].

The growing evidence of the benefits of AAAs has been restricted by the concern that patient safety could be compromised by an increase in the risk of infection acquired from animals, allergic responses, and bites. Brodie et al. have explored this subject in detail in their 2002 review, concluding that in a controlled healthcare environment and with responsible human behavior, the potential benefits far outweigh the risks [38]. The most relevant safety concerns in bringing animals into the pediatric oncology hospital setting are zoonoses and other infections [39]. Patients and Hannibal were closely screened and monitored throughout the study. No known zoonoses were detected. Additionally, no increase in the incidence of other infections was detected in the pre- and post-visit samples. We saw an incidence density (number of events per 100 observed patient days) for fever and infection of about 1 prior to the intervention and 0.6 after the intervention. This is consistent with pre-published results in pediatric oncology. Auletta et al. [40] reported 0.4 to 1.2 events per 100 patient days in a comparable cohort. More recently comparable pre-corona values from the German population were published [41]. Of note, the incidence density was higher prior to the dog visit than after the visit.

Despite a challenging landscape in the inpatient setting, this study highlights that well-defined conditions can be set to facilitate AAAs with visiting dogs without compromising patient safety. The results of this study are consistent with the hypothesis of the beneficial effects of AAAs, with the risks to the patient appearing negligible.

However, the study has several limitations, necessitating further research. The main limitations are the non-controlled, non-randomized design of the study and its small sample size. The sole use of visual analogue face rating scales and unstructured interviews are other limitations. The fact that the interviews were conducted by the dog handler, posed potential additional bias. For a better understanding of the effects of AAAs, the set of qualitative and non-subjective tests would need to be expanded.

In this study, questionnaires and interviews were not aimed at specifically measuring quality of life. Therefore, validated inventories for quality of life, such as the Pediatric Quality of Life Inventory [42], should be obtained from patients, and data regarding quality of life should also be collected from parents and the medical team. Additionally, validated questionnaires on therapy expectations may be administered in future studies. Further studies should also include structured interviews. Microbiology testing would need a better-defined protocol for standardized evaluation. Ideally, the prospective study would be designed as a national or international multicenter trial.

In conclusion, this pilot study provides valuable insights into the introduction of AAIs in the inpatient pediatric oncology setting. The findings suggest that integrating AAAs, particularly with visiting dogs, is a feasible and safe intervention, showcasing promise for enhancing the well-being of pediatric oncology inpatients. However, questions regarding efficacy in quality of life and safety after AAIs warrant a randomized controlled and sufficiently powered prospective study.

## Supplementary Information

Below is the link to the electronic supplementary material.

Supplementary file 1 (PPTX 653 KB)

## Data Availability

The datasets generated during the current study are available from the corresponding author on reasonable request.
