# Supplementary material for: Feasibility, efficacy, and safety of animal-assisted activities with visiting dogs in inpatient pediatric oncology
Source: World J Pediatr. 2024 Aug 7;20(9):915–24. doi: 10.1007/s12519-024-00829-8 (PMC11422466; doi:10.1007/s12519-024-00829-8)
Supplement: Supplementary file 2 — Supplementary file 1 (PPTX 653 KB) [file 12519_2024_829_MOESM1_ESM.pptx]

## Slide 1
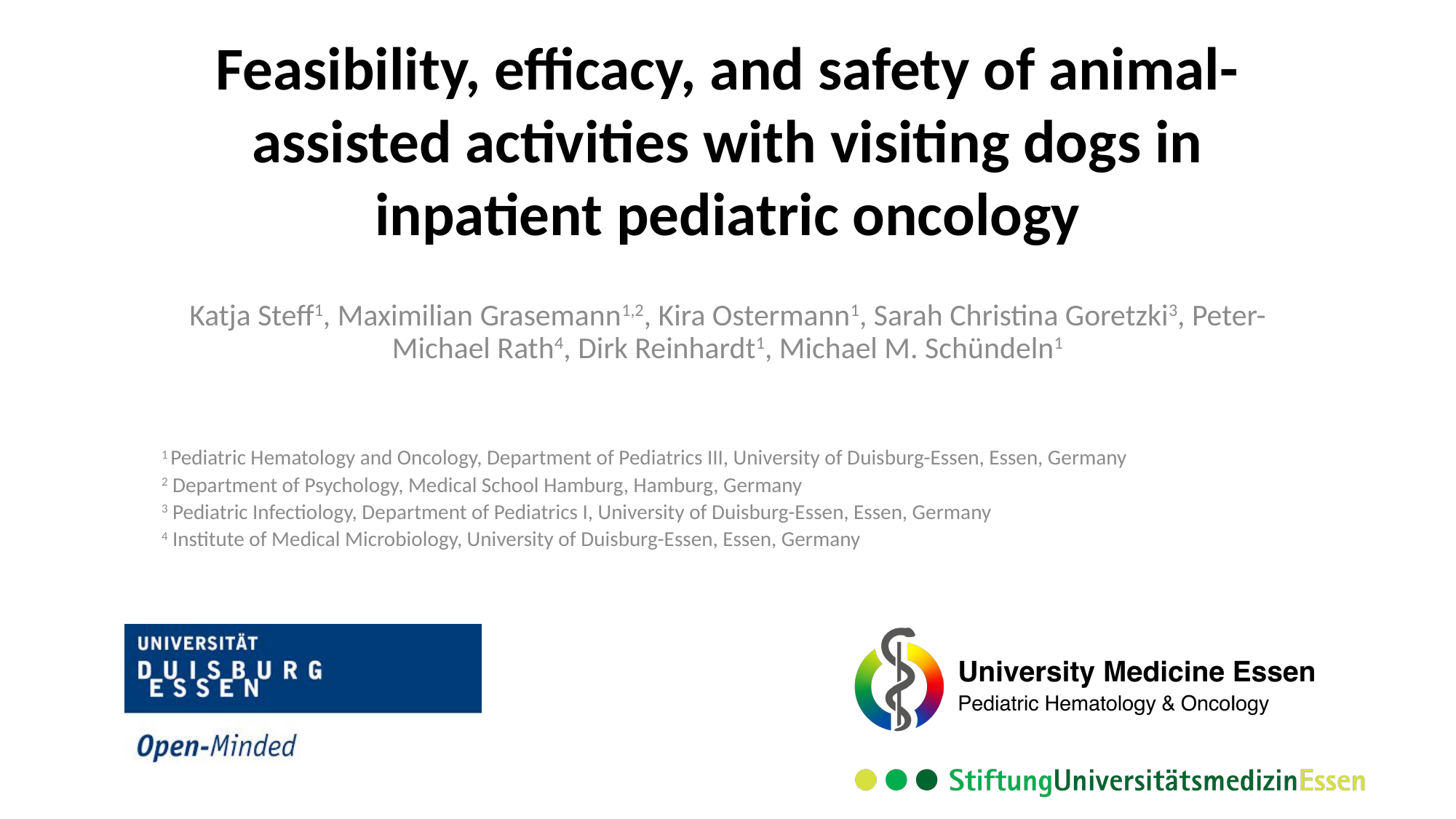

Feasibility, efficacy, and safety of animal-assisted activities with visiting dogs in inpatient pediatric oncology
Katja Steff1, Maximilian Grasemann1,2, Kira Ostermann1, Sarah Christina Goretzki3, Peter-Michael Rath4, Dirk Reinhardt1, Michael M. Schündeln1
1 Pediatric Hematology and Oncology, Department of Pediatrics III, University of Duisburg-Essen, Essen, Germany
2 Department of Psychology, Medical School Hamburg, Hamburg, Germany
3 Pediatric Infectiology, Department of Pediatrics I, University of Duisburg-Essen, Essen, Germany
4 Institute of Medical Microbiology, University of Duisburg-Essen, Essen, Germany
